# Mind the Midstream: Nurse and Patient Perspectives on Diagnostic, Infection Prevention, and Antimicrobial Stewardship for Urinary Tract Infections in Oman

**DOI:** 10.3390/healthcare14213818

**Published:** 2026-11-09

**Authors:** Nawal Al Shizawi, Zaaima Al Jabri, Maria Khan, Areena Hoda Siddiqui, Amal Al Malehi, Zakariya Al Muharrmi, Abdullah Balkhair, Nihal Mohamed Amur Al Riyami, Meher Rizvi

**Affiliations:** 1 Department of Microbiology, Sohar Hospital, Ministry of Health, Sohar 100, Oman; 2 Department of Microbiology and Immunology, College of Medicine and Health Sciences, Sultan Qaboos University, Muscat 123, Oman; 3 Department of Pathology, Peshawar Institute of Cardiology-MTI, Peshawar 25000, Pakistan; kmaria22@hotmail.com; 4 Department of Microbiology, Integral Institute of Medical Sciences, Integral University, Lucknow 226026, India; 5 Department of Family Medicine and Public Health, College of Medicine and Health Sciences, Sultan Qaboos University, Muscat 123, Oman; 6 Department of Microbiology and Immunology, University Medical City, Muscat 112, Oman; 7 Infectious Diseases Unit, Department of Medicine, Sultan Qaboos University Hospital, University Medical City, Muscat 123, Oman; 8 Department of Obstetrics and Gynecology, College of Medicine and Health Sciences, Sultan Qaboos University Hospital, Muscat 123, Oman

**Keywords:** pre-analytic diagnostic stewardship, infection prevention and control, antimicrobial stewardship, UTI

## Abstract

**Background**: Urinary tract infections (UTIs) are a major driver of laboratory testing and antimicrobial prescribing, yet inappropriate urine collection and specimen handling, due to limited patient and nursing engagement, can compromise diagnostic accuracy and contribute to unnecessary or inappropriate antibiotic use. This study evaluated knowledge, attitudes, and practices (KAPs) related to pre-analytical diagnostic stewardship (DS), infection prevention and control (IPC), and antimicrobial stewardship (AMS) amongst nurses and hospitalized patients evaluated for urinary tract infection or undergoing urine culture workup. **Methods**: A cross-sectional study was conducted at two tertiary hospitals in Oman between September 2022 and August 2023. Three structured, internally validated questionnaires assessed pre-analytical DS, IPC, and AMS amongst nurses and patients. A total of 172 nurses were approached, with 165 completing the pre-analytical DS questionnaire and 162 completing the IPC/AMS questionnaire (with overlapping respondents across modules); 77 hospitalized patients evaluated for urinary tract infection or undergoing urine culture workup completed the patient questionnaire (out of 79 initially assessed, with two excluded due to incomplete clinical data). KAP domain scores were standardized to a 0–100 scale and summarized as mean ± SD. MANOVA assessed the effects of covariates on combined KAP outcomes. Mann–Whitney U, Kruskal–Wallis tests compared KAP scores across demographic and professional groups. **Results**: Patients demonstrated strong awareness of the importance of correct urine collection (91%); however, only 7.9% reported receiving midstream urine instructions, contrasting with 86.1% of nurses who reported providing them, pointing to a discordance between nurse-reported practice and patient-reported experience. Patients demonstrated high IPC engagement with 88.3% willing to prompt hand hygiene/glove use among doctors and nurses, and 74% would question antibiotic necessity. Amongst nurses, gaps persisted in inserting new catheters before culture (66.7%) and refrigeration of specimens if delay was expected (36.4%). Work experience significantly influenced nurses’ pre-analytical diagnostic stewardship knowledge and practice scores (*p* = 0.002 for MANOVA global effect; univariate practice score *p* < 0.001). Knowledge amongst nurses was high for hand hygiene, IPC prevention and antibiotic resistance drivers; however, 56.3% incorrectly associated antibiotics with pain/inflammation. Institutional affiliation significantly influenced overall KAP outcomes (*p* = 0.004 for MANOVA global effect, with univariate practice scores showing *p* < 0.001). Overall, findings revealed motivated stakeholders alongside discrete, actionable weaknesses in diagnostic and antimicrobial stewardship workflows. **Conclusions**: This study identifies a critical but modifiable gap between perceived and patient-experienced pre-analytical DS practices in UTIs. Strengthening microbiology and nursing collaborative diagnostic stewardship, standardizing patient instructions, improving specimen-handling infrastructure, and actively engaging patients represent practical, high-impact strategies to enhance diagnostic accuracy, infection prevention, and antimicrobial stewardship in Oman.

## 1. Introduction

Inappropriate antimicrobial prescriptions in urinary tract infections (UTIs) increase the burden of antimicrobial resistance (AMR) [1,2,3,4,5]. Laboratory diagnosis of UTIs continues to be challenging, making it increasingly important to implement diagnostic stewardship (DS). DS optimizes pre-analytical, analytical, and post-analytical aspects of diagnostic testing [6,7]. It is upstream of antimicrobial stewardship (AMS) and plays a vital role in ensuring the right result is released at the right time and providing guidance in appropriate antimicrobial prescribing [8,9,10].

Nurses and patients play an essential role in the execution of the pre-analytic aspect of DS [11,12]. Appropriate sample collection and transport improves the quality of samples, thus reducing unnecessary diagnostic delays, inappropriate treatments and higher costs [13,14]. Diagnostic stewardship (DS), infection prevention and control (IPC), and antimicrobial stewardship (AMS) are deeply interconnected in UTI care. Pre-analytical DS ensures that urine specimens are collected, stored, and transported correctly to yield reliable microbiological results. Simultaneously, IPC practices—including hand hygiene, personal protective equipment (PPE) utilization, and aseptic catheter care—mitigate the risk of healthcare-associated infections. Furthermore, AMS depends on accurate diagnostic data to prevent unnecessary or inappropriate antimicrobial prescribing. Nurses and patients therefore occupy a critical shared interface where diagnostic quality, infection prevention, and antimicrobial decision-making intersect. Few studies have conducted integrated knowledge, attitude and practice (KAP) surveys of nurses and patients in pre-analytic DS, IPC and AMS of UTIs and tailored the educational sessions after analyzing the survey results [15]. Targeted educational sessions for nurses are essential for optimum implementation of DS, AMS and quality improvement [15,16].

Patients are increasingly being considered equal stakeholders in their health outcomes. Involving them in the implementation of IPC, AMS and the pre-analytic aspect of DS may be impactful. An integrated approach that combines DS, IPC, and AMS is vital for strengthening healthcare systems and improving patient safety [17]. This study assessed nurses’ and patients’ KAPs in pre-analytic DS, IPC, and AMS among hospitalized patients evaluated for suspected urinary tract infection or undergoing urine culture workup in order to tailor institutional educational interventions.

## 2. Material and Methods

This cross-sectional study analyzed KAPs related to pre-analytical DS, IPC, and AMS amongst nurses and patients at two tertiary care centers in Oman: Sultan Qaboos University Hospital (facility A), Muscat, and Suhar Hospital (facility B), Suhar, from September 2022 to August 2023. This study was carried out in the Department of Microbiology and Immunology, College of Medicine and Health Sciences (CoMHS), SQU, in coordination with the clinical departments. The study protocol was approved by the institutional ethics committees (unified approval reference: SQU-EC/EC/377/2021; MREC #2678) and the Ministry of Health’s Health Studies and Research Approval Committee (HSRAC; Ref: MoH/CSR/21/24496). For adolescent participants aged 18 years, written informed consent was obtained from parents or legal guardians, along with assent from the participants, in accordance with institutional ethical guidelines. Exclusion criteria comprised patients with severe cognitive impairment, active psychiatric illness precluding interview, or multiple comorbid conditions (defined as the coexistence of ≥3 major chronic systemic illnesses, such as advanced renal failure, decompensated liver cirrhosis, or active malignancy, that would confound data interpretation).

Localized UTI was defined as the presence of localized clinical symptoms (such as dysuria, urinary urgency, frequency, or hematuria), whereas systemic UTI was defined by signs and symptoms of systemic involvement (e.g., fever, chills, delirium, tachycardia, costovertebral angle tenderness, or hypotension); both classifications required concurrent objective laboratory confirmation via urinalysis and urine culture. Patients were eligible if they were hospitalized in the selected wards and had a clinician-documented diagnosis of suspected or confirmed urinary tract infection, catheter-associated urinary tract infection, or a urine culture requested as part of clinical workup or screening (such as pregnancy evaluation or pre-procedural assessment). Nurses posted at and patients with UTIs admitted in medical, surgical, orthopedic, gynecology and obstetrics wards were surveyed through Google Forms. The form contained an initial obligatory consent question, which, if answered with “No”, immediately ended the questionnaire. All questions, except an initial question requesting email address, were obligatory. The final question was followed by links to educational videos on hand hygiene and antimicrobial resistance.

The sample size was determined for the patient cohort based on average monthly medical and surgical ward admissions involving urine culture requests (estimated population of ~200 monthly admissions). Utilizing the Raosoft sample size calculator during study protocol setup (95% CI, 5% margin of error), a minimum required sample size of 63 patient participants was established. Three questionnaires (two for nursing staff and one for patients) were internally validated and adjusted prior to implementation by piloting with a cohort of nurses (*n* = 30) and patients (*n* = 12). The nurses’ questionnaire had two sections each, with 5 sub-sections. The first survey instrument was designed to assess KAPs related to pre-analytical DS (Table 1). It covered the end-to-end workflow, from specimen collection to handling and laboratory transport. The second one assessed their KAPs on the AMS and IPC (Table 1).

**Figure 1:**
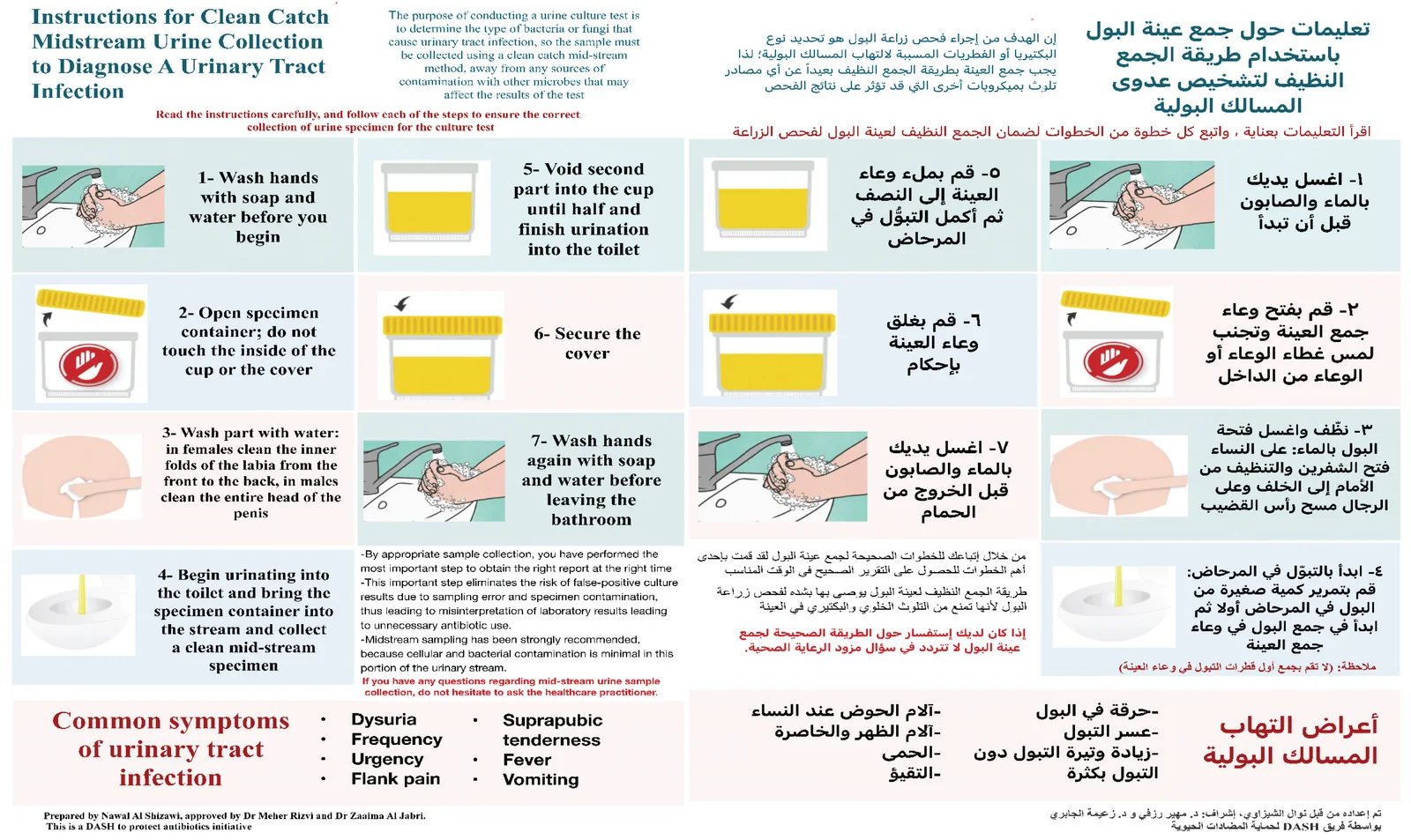
Patient instructions for midstream urine collection (English and Arabic version).

Digital links to the questionnaires were disseminated through ward nurse managers, who facilitated distribution to the nursing staff within their respective units. Participants were sent the link to the form through email and WhatsApp. Educational sessions focused on the DS/AMS/IPC in the context of UTIs. The patients’ questionnaire had 7 sections with the educational moments integrated in the questionnaire itself. Each patient was interviewed individually by trained study personnel. Educational modules and visual instructions were integrated immediately following the relevant questionnaire sections (Table 1). Patients with severe, unstable multi-organ comorbidities, impaired cognitive function, or reduced communication ability were not included, whereas patients with stable underlying chronic conditions (such as controlled hypertension or diabetes) were eligible. Educational modules and visual instructions addressing urine collection techniques, UTI prevention strategies, hand hygiene, patient safety protocols, and basic concepts of AMS were integrated immediately following the relevant questionnaire sections during the individual interviews. Bilingual instructional posters (Arabic and English) detailing clean-catch midstream urine collection techniques were developed and disseminated (Figure 1).

## 3. Statistical Analysis

Raw data sheets were encrypted with VeraCrypt, using Advanced Encryption Standard (AES) with a 256-bit key length (AES-256). Data were analyzed through MS-Excel 365, and descriptive statistics were calculated. Charts of the relevant results were created. The KAP scores were calculated by summing the scores for the corresponding items and transforming them to a scale ranging from 0 to 100, reported as mean ± standard deviation (SD) to align with multivariate linear modeling frameworks. The Kolmogorov–Smirnov test confirmed non-normal distribution across several KAP subdomains (*p* < 0.05). Non-parametric Mann–Whitney U and Kruskal–Wallis tests were conducted to compare score distributions across demographic and professional groups. MANOVA was utilized due to its established robustness to moderate departures from normality in large sample sizes, with Pillai’s trace reported for combined outcomes. To control for false discovery rates across multiple subgroup comparisons, adjusted *p*-values were derived using the Benjamini–Hochberg procedure. Sample size calculations were determined using the Raosoft calculator based on average monthly ward admissions (95% CI, 5% margin of error). A *p*-value less than 0.05 was considered statistically significant. Statistical analyses were conducted using SPSS version 30 (IBM Corp., Armonk, NY, USA).

## 4. Results

A total of 172 nurses were invited to participate. A total of 165 nurses completed the pre-analytical diagnostic stewardship questionnaire, and 162 nurses completed the IPC/AMS questionnaire. Because some nursing staff completed both survey modules while others completed only one, the cohorts represent overlapping rather than strictly disjoint individuals, accounting for minor variations in module-specific totals (as detailed in Supplementary Tables S3 and S6).

## 5. Patient KAPs Regarding Urine Culture Collection Protocols, Infection Control and Antibiotic Usage

A total of 79 patients were assessed for eligibility, of whom 77 patients with complete clinical records were included in the final analysis (two patients were excluded due to missing clinical data fields). Females predominated at 66.2% (51/77).

Questions pertained to DS, and IPC measures (Table 2). A detailed clinical profile is provided in Supplementary Table S1. The majority (66.2%) presented symptoms, with flank pain being the most prevalent (56.9%), often persisting for over a week (45.1% of symptomatic patients). While 66.2% (51/77) of patients presented with acute UTI symptoms, 33.8% (26/77) were asymptomatic, representing patients undergoing urine culture workup for clinical indications such as routine pregnancy screening, pre-procedural evaluation, or hematology workup permitted under this study’s inclusion criteria.

Patients demonstrated a high level of awareness regarding the importance of correct urine specimen collection. Overall, 91% recognized that appropriate sample collection was important for generating accurate microbiological results. However, this awareness was not consistently matched by reported practice. A significant difference in practice was observed between the two hospitals (*p* = 0.008; Supplementary Table S2). Among patients who provided midstream urine samples, only 7.9% reported receiving even cursory instructions from nurses regarding the correct method of collection, while the remainder reported receiving no instructions. This contrasted sharply with nurses’ self-reported practice, where 86.1% stated that they routinely provided clear and complete instructions to patients. This discrepancy highlighted an important gap between provider-reported practice and patient-experienced care. The results did not show a significant difference between Facility A and the comparison hospital. Routine microbiology laboratory surveillance data across the two participating facilities during the study period (September 2022 to August 2023), defining contamination by the isolation of mixed perineal flora or ≥3 organisms from routine urine cultures (facility-wide denominators exceeding 1000 routine cultures per center), indicated contamination rates ranging from 24% to 30%. We hypothesize that this rate may be associated in part with insufficient pre-analytical collection instructions.

Alcohol-based hand rub was the dominant hand hygiene (HH) method (94.8%). Poor adherence (55.8%) to HH was reported amongst relatives and caregivers. PPE was utilized appropriately by healthcare workers (HCWs), as reported by 97.4% of patients. Patients reported that nurses demonstrated good adherence to appropriate IPC practices and PPE usage (78.6%). Following the integration of educational components during the survey process, post-education responses indicated that 88.3% of patients would be willing to prompt healthcare workers to perform hand hygiene and don gloves. Patients were keen to receive guidance on urine sample collection, prevention of recurrence of UTIs, catheter care and the appropriate use of antibiotics supplemented by visual aids.

Only 27.3% of patients were currently on antibiotics, while nearly half (49.4%) had received them previously for UTIs. Compliance with antibiotic prescriptions was high (87%). Additionally, 74.0% indicated an intention to actively question the need for further antibiotic treatment.

## 6. KAPs Among Nurses for Pre-Analytic Diagnostic Stewardship

**Figure 2:**
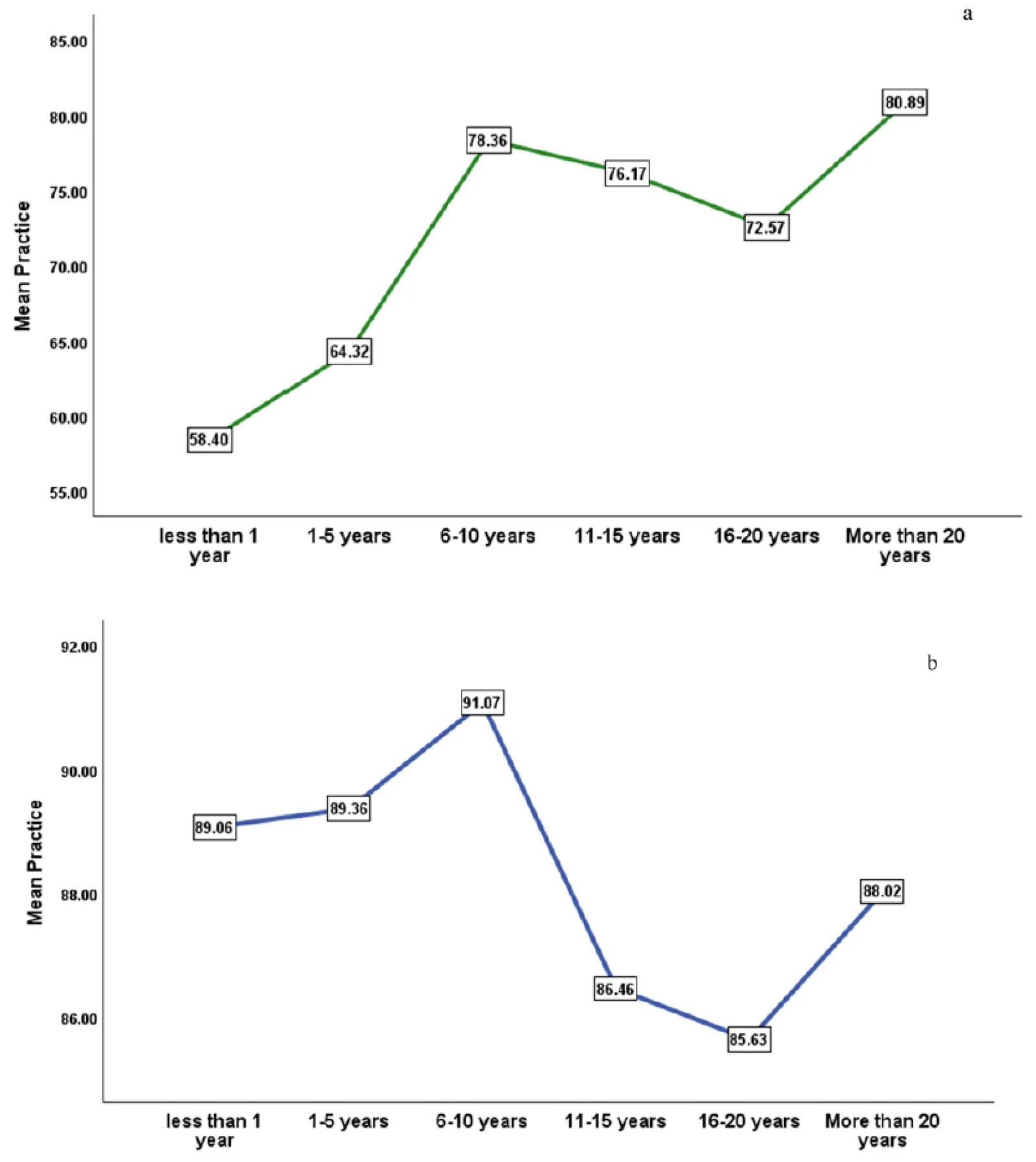
(**a**) Mean practice score of nurses across different levels of work experience in pre-analytic diagnostic stewardship. (**b**) Mean practice score of nurses across different levels of work experience in IPC and AMS. Note: Y-axes are truncated to highlight score variations across experience brackets.

Out of 172 nurses initially approached, 165 completed the pre-analytical DS questionnaire, and 162 completed the IPC/AMS questionnaire (63%: facility A, 37%: facility B), with 29.1% being in the 31–35-year age bracket. Diverse nationalities were noted, with Indian (47.3%) and Omanis (37%) predominating. A significant majority of 72.1% held bachelor’s degrees, and 27.9% possessed 11–15 years’ experience (Supplementary Table S3). On analysis of four variables—institution, gender, highest qualifications and work experience (years)—by MANOVA, work experience showed a significant global effect over the combined dependent variables, KAP (*p* = 0.002), Supplementary Table S4. The results of univariate analysis showed a significant effect of work experience on knowledge (*p* = 0.002) and practice scores (*p* < 0.001). Nurses with 11–15 years’ experience had the highest knowledge score (34.04 ± 8.05), and nurses with 1–5 years’ experience had the lowest knowledge score (25.75 ± 10.04) (Supplementary Table S5). Work experience had a significant impact, both with and without adjustments for other variables (*p* < 0.001). Analysis of the mean practice scores across different levels of work experience revealed a noticeable decline in practice scores between 10 and 20 years of experience. Nurses with more than 20 years of experience achieved the highest mean score of 80.89 ± 11.02 (Figure 2a).

As mentioned above, 86.1% of nurses reported instructing patients on proper urine collection, focusing on avoiding specimen contamination (89.9%) and collection of a clean midstream sample (MSU) (88.1%). Regarding guidance, 63.6% (105/165) reported that some form of guidance for proper urine collection was available, with verbal instructions reported by 42.9% and written instructions by 30.5% (32/105). Most (88.5%) believed that their duties extended to instructing patients on appropriate collection. Most (84.8%) had received training in sample collection from catheterized patients; 88.5% obtained the specimen via the catheter tube, 87% disinfected the sampling port prior to collection, and 66.7% replaced catheters in place >2 weeks before culture. Patient education was considered important by 84.2% for accurate microbiology results. Immediate sample transport (74.5%) was practiced, but if delays occurred, refrigeration was not universal (36.4% reported refrigeration; 29.7% lacked access). Our educational initiatives were welcomed by 86.1% of nurses, with 77.6% (128/165) expressing a desire for educational information on the topic, with preferences distributed among poster-based education (40.6%) and in-person presentations (39.4%) (Table 3).

## 7. KAPs Among Nurses for IPC and AMS

Amongst the 162 nurses who responded, 98.8% understood the importance of HH before/after patient contact and sample handling (Table 4). Universal agreement existed on IPC’s crucial role and HH importance (98.8%) in preventing infections. Nosocomial infections were correctly identified by 94.4%, and 98.8% understood their link with adverse patient outcomes. The majority, 97.5%, were aware that antibiotic utility extended to treating bacterial infections. Additionally, 56.3% of participants incorrectly identified antimicrobials as treatments for pain and inflammation. Exceptional knowledge of side effects (allergic reactions 93.1%, common symptoms 96.3%) and impact on endogenous flora (80.6%) was observed. Excellent awareness of reasons behind the escalating antimicrobial resistance (inappropriate prescribing and dosage (94.4%), loss of activity of antibiotics against bacteria (94.4%), use of broad-spectrum antibiotics (79.6%), and poor IPC (77.8%) was demonstrated.

The importance of IPC and AMS interventions in curbing AMR and optimum patient outcome was appreciated by 88.9% of nurses. MANOVA revealed that the institutional culture had a significant global effect on the combined dependent variable, KAP (*p* = 0.004) (Supplementary Table S4), which was explored further by univariate analysis, with and without adjusting for the effect of other variables. The practice scores revealed that the facility itself had a highly significant effect (*p* < 0.001). Facility B had a higher practice score (90.67 ± 6.28) than Facility A (Supplementary Table S6). Without adjusting for other variables, work experience appeared to have a significant effect (*p* = 0.04); however, after adjusting, no significance was observed (*p* = 0.09). Assessing the meaning of the practice score within the years of work experience, the practice score increased until 10 years (the highest mean score, 91.07 ± 5.31) but declined after 10 years (Figure 2b).

## 8. Discussion

This study recognizes that both nurses and patients are important stakeholders in optimizing patient care, and tailored continuous education is essential to achieve this. Carefully structured KAPs are useful tools to understand baseline competencies and help identify which areas need augmented training and where positive feedback would be promoting their engagement and ownership.

Effective DS hinges entirely on the excellent execution of the pre-analytic component, the primary players of which are the nurses and the patients themselves. It was heartening to find that patients clearly understood the importance of a correctly collected sample, underscoring a high awareness of causality [10,18]. Patient involvement shifts their role from passive recipients to active co-producers of care [19]. This engagement reduces preventable errors and optimizes patient management. Our study uniquely engages patients in improving DS and AMS in UTIs, an initiative which revealed a gap in the nurses’ knowledge and practice regarding instructions provided in the collection of MSU. Over 90% of patients reported a lack of guidance, as against 86% of nurses who reported providing the instructions. This highlights an area for improvement. This contrasts with Eley et al.’s (2016) finding where 50% received instructions [20]. Providing both verbal and written instructions has been linked to better patient knowledge and satisfaction [20]. Incomplete or insufficient instructions identified in our findings may be hypothesized to contribute to the routine laboratory-surveillance contamination rates (24–30%) observed across the two facilities, highlighting potential gaps in educational methods and delivery from the patient’s perspective. Lack of clear instruction leads to contamination and false-positive results, thus impacting DS [13]. Inculcating best practices amongst nurses through continuous education will strengthen pre-analytic DS and curb false positive results and inappropriate antibiotic use [21]. While 66.2% of patients presented with acute urinary symptoms, 33.8% were asymptomatic, which included clinical indications such as pregnancy screening or pre-procedural workups. Rather than viewing these as false positives or attributing them directly to physician ordering errors (as physicians were not surveyed), these findings highlight the clinical presence of asymptomatic bacteriuria. Distinguishing true infection from asymptomatic colonization remains an important opportunity for future diagnostic stewardship interventions.

A majority (78.6%) of catheterized patients reported adherence to HH and PPE use during catheter insertion, indicating good IPC practices. Other studies have reported similar findings [22]^.^ Among the subset of catheterized patients (*n* = 14), 71.4% reported understanding daily questioning regarding catheter necessity. The relatively flat hierarchy in Oman empowers patients (88.3%) to unhesitatingly request HH or don gloves prior to examination. This contrasts with steep vertical hierarchies in many countries [23].

An interesting observation in our findings is the contrast between high patient-reported rates of visible universal precautions (such as PPE use at 97.4%) and lower aggregate composite practice scores (ranging from 15 to 51 out of 100). This divergence likely reflects that patients readily observe tangible barrier precautions while being less positioned to evaluate nuanced pre-analytical practices, timing of hand hygiene, or verbal instructions. Appropriate treatment duration depends on the clinical syndrome, antimicrobial agent, severity of infection, and patient characteristics. Most patients (87%) reported adherence to completing antibiotic courses, a practice which aligns with the current guidance [24]. Interestingly, 74% stated they would inquire why antibiotics were prescribed, which serves as an excellent nudge to the doctors to rethink the prescription. This receptivity among patients underscores the potential value of patient-centered health literacy in supporting antimicrobial stewardship, which can be further strengthened through targeted visual and verbal education [25]. However, decisions regarding antimicrobial selection and treatment duration depend on the clinical syndrome, severity of illness, microbiological findings, and patient-specific factors. While patient empowerment, such as encouraging individuals to inquire about the necessity and expected duration of therapy, is beneficial, it must be balanced with reinforcing adherence to evidence-based, clinician-prescribed regimens.

Since routine urine samples are usually MSU, the nurses’ role in guiding patients in collection of appropriate urine samples is crucial but not fully appreciated by them. Mere availability of guidelines will not suffice, as only 63.6% were aware of it. This reinforces the role of active training. A significant discordance was identified between nurse-reported practice and patient-reported experience regarding the provision and clarity of midstream urine collection instructions. This underscores the importance of regular in-person training in the pre-analytic component of DS. We believe that DS in MSU needs to be urgently spearheaded, as it remains a severely neglected area. The nurses (88.5%) accepted their responsibility to educate patients and understood that poor collection leads to false reports and unnecessary treatment [26]. Aligning with CDC guidelines (2019), the majority of them (66.7%) recognized the necessity of replacing indwelling catheters that had been in situ for more than two weeks prior to specimen collection [27]. In terms of sample handling, a concerning percentage (29.7%) lacked refrigeration access and needs addressing [9,28]. Although most nurses reported appropriate catheter urine sampling practices, the identified gaps remain clinically significant. Suboptimal port disinfection, sampling from downstream collection bags, or failure to replace long-standing catheters before sampling can introduce specimen contamination, potentially contributing to erroneous microbiological interpretation and secondary antimicrobial overexposure.

Our study revealed that work experience significantly influenced pre-analytic DS knowledge (*p* = 0.002) and practice scores (*p* < 0.001).

This suggests that pre-analytic DS competencies evolve alongside clinical experience and professional development, exhibiting non-linear variations across career stages rather than a strictly monotonic increase. These findings highlight the importance of structured mentoring and continuous professional training to improve specimen quality and diagnostic accuracy. Studies have shown that pre-analytical DS directly influences microbiological results, antimicrobial prescribing, and patient outcomes [29].

Overall, the nurses demonstrated sound IPC awareness. The increasing concern of AMR in the Middle East highlights the importance of implementing IPC practices [26]. Our study found high HH adherence rates among nurses (98.8% during patient care, 95% during sample handling), comparable to other centers [30]. A study reported a 79.6% HH compliance rate among nurses [31], while Al Dawsari et al. reported 55.8% compliance amongst nurses [32]. Nurses were well conversant with the antimicrobial spectrum (97.5%) and appreciated the ensuing collateral damage (80.6%). A significant majority (90%) correctly identified that inappropriate dosing, misuse, and the over-reliance on broad-spectrum agents are key drivers of antimicrobial resistance. They also acknowledged that poor IPC practices promote AMR [33]. While the majority of participants still believed it important to complete the antibiotic course (>90%) and advised patients accordingly (96.9%), this traditional practice contrasts with modern antibiotic stewardship guidelines, such as the EAU Guidelines, which favor shorter, highly targeted treatment durations to minimize resistance risk [24]. Most nurses (88.8%) believed that AMS and DS initiatives improve patient outcomes. Such a high level of awareness amongst nurses is essential to promote AMS. Significant institutional differences in IPC and AMS practice domains were observed (adjusted *p* < 0.001), suggesting greater institutional commitment and multidisciplinary collaboration play a big role in improvement [34,35]. Because these operational variables were not directly measured in the present study, the observed facility-level differences may reflect variations in local workflows, training practices, availability of written instructions, staffing patterns, or specimen-handling infrastructure. These potential associations warrant further evaluation in future implementation research.

Our findings indicate that continuous competency-based training in diagnostic stewardship, IPC, and AMS is essential for nursing staff across all career stages, with particular emphasis on supporting early-career nurses and providing career-long professional development, as practice scores demonstrated non-linear variation across experience brackets. To address these gaps, actionable strategies should focus on (1) developing standardized, bilingual pictorial instructions for midstream urine collection; (2) implementing patient ‘teach-back’ methods prior to specimen submission; (3) introducing standardized catheter sampling checklists; (4) establishing clear cold-chain workflows and rapid transport mechanisms (or using preservative tubes) when transport delays occur; and (5) providing regular microbiology surveillance feedback to clinical wards regarding specimen rejection and contamination rates.

Based on nurses’ expressed interest, a comprehensive symposium on antimicrobial and diagnostic stewardship and IPC was organized, involving all relevant stakeholders (nurses, physicians, lab technicians and pharmacists), an interdisciplinary approach which we strongly advocate. This broader approach assesses the KAPs and then educates the stakeholders, thus promoting multimodal diagnostic and antimicrobial stewardship [28,36]. In future surveys or multicenter analyses, we will statistically adjust for center-level variability and place greater focus on the direct interaction and communication between individual patients and nurses.

## 9. Limitations

This study has several limitations. First, participants were convenience-sampled via ward managers and WhatsApp across two tertiary centers, resulting in an unknown response rate for the IPC/AMS survey and limiting generalizability beyond the two participating centers. Second, the nurse and patient cohorts represent unlinked samples from distinct clinical encounters and shifts. Third, data collection relied on self-reported practices, making responses susceptible to social desirability bias (potentially leading to over-reporting of optimal IPC/AMS behaviors), recall limitations, and interviewer bias, particularly given that study personnel administered questionnaires alongside embedded educational initiatives. Fourth, the survey instruments lacked formal psychometric reliability and validity testing prior to deployment. Finally, patient KAP responses regarding infection control and stewardship were actively influenced by the embedded educational components integrated during survey administration rather than representing pure baseline knowledge.

## 10. Conclusions

This KAP spotlights a critical need: strengthening the pre-analytic phase of diagnostics, especially collection and handling of MSU. Significant gaps in knowledge and practices among both nurses and patients have direct implications for diagnostic accuracy, patient safety, and antimicrobial stewardship. While attitudes were generally positive, inconsistent adherence to evidence-based practices such as proper urine collection and timely transport undermines the effectiveness of diagnostic stewardship initiatives.

This study highlights an apparent discordance between nurse-reported provision of midstream urine collection instructions and patient-recollected receipt of such guidance. Given that these estimates derive from unpaired cohorts and may be influenced by patient recall limitations, social desirability bias, and specimen collection in acute triage settings prior to ward admission, this finding should be interpreted cautiously as an indicator of potential communication gaps rather than direct procedural failure. Nurses, being the first point of contact in clinical workflows, play a pivotal role in ensuring that diagnostic processes are optimized. Their empowerment through ongoing, career-long training in IPC, sample handling, and the principles of antimicrobial resistance mitigation is vital across all stages of professional experience. Simultaneously, patient education campaigns must be developed to encourage compliance with hygiene, sampling, and follow-up protocols.

Enhancing the diagnostic literacy and strengthening the pre-analytical phase through stewardship-informed strategies is not merely a laboratory concern; it is a public health imperative. Robust implementation of DS, AMS and IPC programs is essential across all healthcare institutions and recommended to stem the tide of AMR.

## Figures and Tables

**Table 1:** KAP questionnaire categories among patients and nurses.

Section	Patients’ Questionnaires Sample Handling, IPC, Antimicrobial Stewardship (ASP)	Nurses’ Questionnaire-1 Pre-Analytic Diagnostic Stewardship for Urine Culture and Sensitivity.	Nurses’ Questionnaire-2 IPC, HAI, ASP
1.	Informed consent	Informed consent	Informed consent
2.	Demographic characteristics	Baseline demographic and work-related variables	Demographics and work-related information
3.	Clinical profile and medical characteristics	Patient preparation for urine sample collection	Prevention and control of healthcare-associated infections
4.	Patient’s practice and actual preparation for urine sample collection	Urine sample handling procedures and transportation to the laboratory	Appropriate antibiotic use, antibiotic resistance and prevention of antibiotic resistance
5.	Infection prevention and control	Feedback and opinion	Feedback and opinion
6.	Knowledge about appropriate antibiotic use and antimicrobial resistance		
7.	Feedback and opinion		

**Table 2:** Clinical profile of patients and their KAPs for re-analytic diagnostic stewardship (*n* = 77).

Domain	Key Finding	*n*/*N* (%)
Clinical presentation	Patients presenting with UTI symptoms	51 (66.2%)
	Flank pain among symptomatic patients	29/51 (56.9%)
	Symptoms lasting >1 week among symptomatic patients	23/51 (45.1%)
Comorbidities (*n* = 77)	Patients with ≥1 comorbidity	60 (77.9%)
	Hypertension	25/77 (32.5%)
	Diabetes mellitus	24/77 (31.2%)
	Dyslipidemia	11/77 (14.3%)
	Renal stone	9/77 (11.7%)
	No comorbidity	17/77 (22.1%)
MSU collection (non-catheterized, *n* = 63)	Received instructions on MSU collection	5/63 (7.9%)
	Instructions provided by nurse	5/5 (100%)
Catheter-related practices (catheterized, *n* = 14)	Reported hand hygiene/glove use before procedures	11/14 (78.6%)
	Observed urine collected from catheter tube	8/14 (57.1%)
	Believed daily questioning of catheter necessity is important	10/14 (71.4%)
	Wanted education on catheter care before discharge	14/14 (100%)
Perception of diagnostic accuracy (all participants, *n* = 77)	Believed correct sample collection improves microbiology result accuracy	70/77 (90.9%)

**Table 3:** Nurses’ knowledge, attitudes, and practices regarding urine culture pre-analytical diagnostic stewardship and collection guidance.

Survey Item/Indicator	Number (*n*)	Total Valid Responses (*N*)	Percentage (%)
Institutional Guidance and Protocols			
Any form of guidance/protocol available for MSU collection	105	165	63.60%
Written instructions available in the ward	32	105	30.50%
Verbal guidance routinely provided to patients	71	165	42.90%
Patient Education and Instruction Practices			
Nurse considers patient instruction as part of their professional duty	146	165	88.50%
Routinely instructs patients on midstream clean-catch technique	142	165	86.10%
Instructions include specific steps to avoid perineal skin contamination	127	142	89.40%
Considers patient education vital for accurate microbiology results	139	165	84.20%
Catheterized Specimen Collection Practices			
Formal training received on catheterized urine collection	140	165	84.80%
Obtains specimen directly from catheter sampling port	146	165	88.50%
Disinfects sampling port prior to specimen collection	143	165	86.70%
Replaces catheter in place >2 weeks prior to obtaining culture	110	165	66.70%
Specimen Handling and Transport			
Immediate transport of specimen to laboratory (<2 h)	123	165	74.50%
Refrigerates specimen if transport delay occurs	60	165	36.40%
Lacks access to refrigeration facilities for delayed samples	49	165	29.70%
Educational Preferences and Needs			
Welcomes departmental educational initiatives on diagnostic stewardship	142	165	86.10%
Desires further educational information on pre-analytical stewardship	128	165	77.60%
Preferred delivery format: Poster-based educational materials	63	155	40.60%
Preferred delivery format: In-person clinical training/presentations	61	155	39.40%

**Table 4:** Summary of knowledge, attitudes and practices (KAPs) regarding healthcare-associated infections, antimicrobial use and antimicrobial stewardship among nurses (*n* = 162).

Domain	Key Indicator	Number (*n*)	Total (*N*)	Percentage (%) *
Knowledge—IPC	Correctly reported hand hygiene performance before and after patient contact	160	162	98.80%
	Agreed IPC is key to preventing infection spread	162	162	100.00%
	Agreed effective handwashing is most important for infection prevention	162	162	100.00%
	Correctly recognized nosocomial infections are acquired in healthcare settings	153	162	94.40%
	Agreed HAIs increase morbidity, mortality, and hospital stay	160	162	98.80%
Knowledge—AMR	Recognized that antibiotics are used to treat bacterial infections	158	162	97.50%
	Agreed inappropriate antibiotic use promotes AMR	153	162	94.40%
	Agreed broad-spectrum antibiotic use increases AMR	129	162	79.60%
	Agreed poor infection control contributes to spread of AMR	126	162	77.80%
	Agreed improved healthcare hygiene reduces spread of resistant organisms	160	162	98.80%
	Recognized narrow-spectrum therapy following culture testing reduces AMR	137	162	84.60%
	Believed AMR will become a greater clinical problem in the future	119	162	73.50%
Practice/Stewardship	Agreed nurses should review microbiology culture reports and inform clinicians	110	162	67.90%
	Agreed diagnostic and antimicrobial stewardship improves clinical outcomes	144	162	88.90%
	Had received formal antimicrobial stewardship training	33	162	20.40%
	Supported educational activities on infection prevention and control	150	162	92.60%
	Believed all healthcare professionals should receive AMR training	151	162	93.20%

* Percentages calculated on respondents only.

## Data Availability

The original contributions presented in this study are included in the article/Supplementary Material. Further inquiries can be directed to the corresponding author.

## Supplementary Materials

The following supporting information can be downloaded at: https://www.mdpi.com/article/10.3390/healthcare14213818/s1, Table S1: Clinical profile of patients; Table S2: Comparison of Patient-Reported Staff Practice Scores Across Hospitals According to Patient Educational Level (N = 77) **; Table S3: Demographic characteristics of the participating nurses; Table S4: MANOVA analysis of four variables in the pre-analytic diagnostic stewardship, IPC, AMS, and KAP; Table S5: Analysis of KAPs of the nurses’ pre-analytic diagnostic stewardship for urinary tract infections; Table S6: Nurses’ assessment of knowledge, awareness, and practice for prevention and control of healthcare-associated infections and antimicrobial stewardship.
