# Supplementary material for: Mind the Midstream: Nurse and Patient Perspectives on Diagnostic, Infection Prevention, and Antimicrobial Stewardship for Urinary Tract Infections in Oman
Source: Healthcare (Basel). 2026 Nov 9;14(21):3818. doi: 10.3390/healthcare14213818 (PMC13762842; doi:10.3390/healthcare14213818)
Supplement: Supplementary file 1 [file healthcare-14-03818-s001.zip › Supplementary Table S1.pdf]

Supplementary Table S1: Clinical Profile of Patients

Characteristic	Value/Category	n (%)	Characteristic	Value/Category	n (%)
Total Patients Assessed		79	Symptom Status	Symptomatic	51 (66.2%)
Total Analyzed Cohort		77		Asymptomatic	26 (33.8%)
SQUH		44 (57.1%)	Symptoms (among symptomatic, n=51)	Flank pain	29 (56.9%)
Suhar Hospital		33 (42.9%)		Fever	24 (47.1%)
Gender (n=77)	Female	51 (66.2%)		Burning micturition	22 (43.1%)
	Male	26 (33.8%)		Frequency	17 (33.3%)
Admission Ward	Female surgical	7 (9.1%)		Nausea	15 (29.4%)
	Male surgical	12 (15.6%)		Vomiting	15 (29.4%)
	Female medical	17 (22.1%)		Urgency	13 (25.5%)
	Male medical	6 (7.8%)		Urinary incontinence	13 (25.5%)
	Obstetrics/Gynae	13 (16.9%)		Urine retention	10 (19.6%)
	Maternity	6 (7.8%)		Foul smelling urine	11 (21.6%)
	Orthopedics/Neuro	5 (6.5%)		Hematuria	8 (15.7%)
	Hematology	10 (13.0%)	Symptom Duration	< 1 week	17 (33.3%)
	Other	1 (1.3%)		1 week	11 (21.6%)
Country of Origin	Oman	73 (94.8%)		> 1 week	23 (45.1%)
	Sudan/Tanzania	4 (5.2%)	Comorbidities (n=77)	Patients with ≥1 comorbidity	60 (77.9%)
Age Group (Years)	11–20	7 (9.1%)		Hypertension	25 (32.5%)
	21–30	17 (22.1%)		Diabetes mellitus	24 (31.2%)
	31–40	20 (26.0%)		Dyslipidemia	11 (14.3%)
	41–50	7 (9.1%)		Renal stone	9 (11.7%)
	51–60	4 (5.2%)		Other Comorbidities	0 (0.0%)

	> 60	22 (28.6%)		No comorbidity	17 (22.1%)
Level of Education	Primary	5 (6.5%)	Catheter Status	Catheterized	14 (18.2%)
	Secondary	8 (10.4%)		Non-catheterized	63 (81.8%)
	High School/Diploma	24 (31.2%)	Marital Status	Single	19 (24.7%)
	Higher Diploma	4 (5.2%)		Married	51 (66.2%)
	Bachelor	11 (14.3%)		Widowed	7 (9.1%)
	PhD/Other	2 (2.6%)			
	Uneducated	23 (29.9%)			

Note*:* A total of 79 patients were initially approached, of whom 77 patients with complete clinical datasets were included in the final analysis (2 patients had missing clinical data fields and were excluded from clinical variable tabulations). All percentages for clinical characteristics, symptoms, and comorbidities are calculated using $n = 77$ as the denominator.
