# Supplementary material for: Mind the Midstream: Nurse and Patient Perspectives on Diagnostic, Infection Prevention, and Antimicrobial Stewardship for Urinary Tract Infections in Oman
Source: Healthcare (Basel). 2026 Nov 9;14(21):3818. doi: 10.3390/healthcare14213818 (PMC13762842; doi:10.3390/healthcare14213818)
Supplement: Supplementary file 1 [file healthcare-14-03818-s001.zip › Supplementary Table S2.pdf]

Supplementary Table S2: Comparison of Patient-Reported Staff Practice Scores Across Hospitals According to Patient Educational Level ($N = 77$)**

Educational Level	Hospital	Sample Size (N)	Mean Score ± SD	*p*-value*
Uneducated	Sohar Hospital	9	31.00±17.92	0.43
	Sultan Qaboos University Hospital	14	50.63±26.45	
Secondary / Diploma	Sohar Hospital	21	30.00±23.66	0.19
	Sultan Qaboos University Hospital	20	41.00±25.73	
Bachelor's and Above	Sohar Hospital	4	15.00±19.14	0.15
	Sultan Qaboos University Hospital	9	40.00±26.19	

*Mann-Whitney Test

** The patients have been asked five questions regarding the performance of doctors and nurses in training and collecting samples.
