# Supplementary material for: Mind the Midstream: Nurse and Patient Perspectives on Diagnostic, Infection Prevention, and Antimicrobial Stewardship for Urinary Tract Infections in Oman
Source: Healthcare (Basel). 2026 Nov 9;14(21):3818. doi: 10.3390/healthcare14213818 (PMC13762842; doi:10.3390/healthcare14213818)
Supplement: Supplementary file 1 [file healthcare-14-03818-s001.zip › Supplementary Table S3.pdf]

Supplementary Table S3: Demographic characteristics of the participating Nurses

Characteristic	Value/Category	n (%)	Characteristic	Value/Category	n (%)
	Institution	165		21-25	11 (6.7%)
	SQUH	104 (63%)		26-30	41 (24.8%)
**Total Participants**	Suhar Hospital	61 (37%)		31-35	48 (29.1%)
	Female	129 (78.2%)		36-40	34 (20.6%)
**Gender**	Male	36 (21.8%)		41-45	14 (8.5%)
	Omani	61 (37%)		46-50	7 (4.2%)
	Indian	78 (47.3%)	**Age Group (Years)**	>50	10 (6.1%)
	Filipino	22 (13.3%)		Diploma	34 (20.6%)
				Advanced Diploma	4 (2.4%)
				Bachelor	119 (72.1%)
**Nationality**	Other	4 (2.4%)	**Highest Qualification**	Master	8 (4.8%)
	< 1	10 (6.1%)			
	1-5	38 (23%)			
	6-10	39 (23.6%)			
	11-15	46 (27.9%)			
	16-20	14 (8.5%)			
**Work Exp.(Years)**	>20	18 (10.9%)			
