# Supplementary material for: Mind the Midstream: Nurse and Patient Perspectives on Diagnostic, Infection Prevention, and Antimicrobial Stewardship for Urinary Tract Infections in Oman
Source: Healthcare (Basel). 2026 Nov 9;14(21):3818. doi: 10.3390/healthcare14213818 (PMC13762842; doi:10.3390/healthcare14213818)
Supplement: Supplementary file 1 [file healthcare-14-03818-s001.zip › Supplementary Table S4.pdf]

Supplementary Table S4: MANOVA analysis of four variables in the Pre-analytic Diagnostic Stewardship and IPC and AMS KAP

**MANOVA analysis of four variables in the Pre-analytic Diagnostic Stewardship KAP**			
**Variables**	**Wilks' Lambda**	**F**	**P-value**
Institution	0.99	0.32	0.81
Gender	0.96	2.40	0.07
Highest Qualification	0.95	0.91	0.52
Work Experience (Years)	0.80	2.43	0.002
**MANOVA analysis of four variables in IPC & AMS**			
**Variables**	**Wilks' Lambda**	**F**	**P-value**
Institution	0.91	4.69	0.004
Gender	0.98	0.86	0.46
Highest Qualification	0.94	1.07	0.38
Work Experience (Years)	0.88	1.28	0.21
