# Supplementary material for: Mind the Midstream: Nurse and Patient Perspectives on Diagnostic, Infection Prevention, and Antimicrobial Stewardship for Urinary Tract Infections in Oman
Source: Healthcare (Basel). 2026 Nov 9;14(21):3818. doi: 10.3390/healthcare14213818 (PMC13762842; doi:10.3390/healthcare14213818)
Supplement: Supplementary file 1 [file healthcare-14-03818-s001.zip › Supplementary Table S5.pdf]

Supplementary Table S5: Analysis of KAP of the Nurses' Pre-Analytic Diagnostic Stewardship for Urinary Tract Infections

		N	Knowledge	Attitude	Practice
**Total**		166	31.20 ± 9.89	63.40 ± 8.32	73.11 ± 16.61
**Institution**	SQUH	105	32.18 ± 10.01	63.77 ±7.70	74.40 ± 15.63
	Suhar Hospital	61	29.51 ± 9.54	62.75 ± 9.31	70.89 ± 18.10
	**P-value**		**0.74**	**0.26**	**0.25**
	Adjusted P-value		0.93	0.58	0.43
**Gender**	Female	129	30.90 ± 9.91	63.44± 8.17	74.48 ±15.71
	Male	37	32.24 ± 9.91	63.24 ± 8.94	68.32 ± 18.91
	**P-value**		**0.54**	**0.87**	**0.09**
	Adjusted P-value		0.61	0.93	0.04
**Highest Qualification**	Diploma	34	33.40 ± 7.81	64.59 ± 5.31	77.65 ± 11.66
	Advanced Diploma	4	23.21 ± 8.99	61.0 ± 2.0	79.0 ± 9.45
	Bachelor	120	30.71 ± 10.25	63.20 ± 9.04	71.70 ± 17.66
	Master	8	33.04 ± 11.41	62.50 ± 9.78	72.0 ± 19.24
	**P-value**		**0.17**	**0.67**	**0.45**
	Adjusted P-value		0.19	0.71	0.83
**Work Exp.(Years)**	< 1	10	27.14 ± 9.99	59.20 ± 12.19	58.40 ± 18.78
	1-5	38	25.75 ± 10.04	62.32 ± 11.11	64.32 ± 19.0
	6-10	39	31.50 ± 9.93	64.62 ± 7.92	78.36 ± 12.40
	11-15	47	34.04 ± 8.05	64.0 ± 7.08	76.17 ± 13.32
	16-20	14	33.16 ± 8.22	62.57 ± 3.37	72.57 ± 20.02
	>20	18	35.32 ± 10.53	64.44 ± 4.53	80.89 ± 11.02
	**P-value**		**<0.001**	**0.32**	**<0.001**
	**Adjusted P-value**		**0.002**	**0.57**	**<0.001**
