# Supplementary material for: Mind the Midstream: Nurse and Patient Perspectives on Diagnostic, Infection Prevention, and Antimicrobial Stewardship for Urinary Tract Infections in Oman
Source: Healthcare (Basel). 2026 Nov 9;14(21):3818. doi: 10.3390/healthcare14213818 (PMC13762842; doi:10.3390/healthcare14213818)
Supplement: Supplementary file 1 [file healthcare-14-03818-s001.zip › Supplementary Table S6.pdf]

Supplementary Table S6: Nurses' Assessment of Knowledge, Awareness, and Practice for Prevention and Control of Healthcare-Associated Infections and Antimicrobial Stewardship

		N	Knowledge	Attitude	Practice
**Total**		162	84.87 ± 7.32	90.05 ± 13.37	88.31 ± 7.67
**Institution**	SQUH	87	84.95 ± 6.82	89.22 ±12.95	86.28 ± 8.19
	Suhar Hospital	75	84.78 ± 7.91	91.0 ± 13.87	90.67 ± 6.28
	**P-value**		**0.75**	**0.21**	**<0.001**
	Adjusted P-value		0.60	0.78	**<0.001**
**Gender**	Female	142	84.66 ± 7.40	89.61± 13.48	88.56 ±7.63
	Male	20	86.34 ± 6.73	93.13 ± 12.48	86.56 ± 7.93
	P-value		0.36	0.17	0.20
	Adjusted P-value		0.40	0.34	0.35
**Highest Qualification**	Diploma	46	85.79 ± 6.57	90.22 ± 12.61	88.73 ± 8.29
	Advanced Diploma	4	88.42 ± 2.34	93.75 ± 7.22	89.06 ±5.98
	Bachelor	109	84.20 ±7.72	90.02 ± 13.90	88.07 ±7.54
	Master	3	90.24 ±2.44	83.33 ± 14.43	89.58 ± 7.22
	P-value		0.25	0.76	0.86
	Adjusted P-value		0.16	0.50	0.72
**Work Exp.(Years)**	< 1	4	87.20 ± 4.62	96.87 ± 6.25	89.06 ± 5.98
	1-5	37	84.51± 8.82	90.88 ± 14.63	89.36 ± 7.04
	6-10	35	83.83 ± 6.75	89.64 ± 11.93	91.07 ± 5.31
	11-15	42	85.48 ± 6.96	92.26 ± 12.94	86.46 ± 9.56
	16-20	20	86.46 ± 4.07	88.13 ± 11.81	85.63 ± 7.34
	>20	24	84.15 ± 8.78	85.94 ± 15.77	88.03 ± 7.35
	**P-value**		**0.77**	**0.27**	**0.04**
	Adjusted P-value		0.71	0.43	**0.09**
